# Guava Leaf Extract Diminishes Hyperglycemia and Oxidative Stress, Prevents *β*-Cell Death, Inhibits Inflammation, and Regulates NF-kB Signaling Pathway in STZ Induced Diabetic Rats

**DOI:** 10.1155/2018/4601649

**Published:** 2018-02-18

**Authors:** Muthukumaran Jayachandran, Ramachandran Vinayagam, Ranga Rao Ambati, Baojun Xu, Stephen Sum Man Chung

**Affiliations:** ^1^Food Science and Technology Program, Beijing Normal University-Hong Kong Baptist University United International College, Zhuhai, Guangdong 519087, China; ^2^Department of Biotechnology, Vignan's Foundation for Science, Technology and Research (Deemed to Be University), Vadlamudi, Guntur, Andhra Pradesh 522213, India

## Abstract

Traditional Chinese medication has been utilized by Chinese medical practitioners to treat the varied symptoms of diabetes mellitus (DM). Notably, guava leaf has been used to treat diabetes in Asia. Our present study has been designed to analyze the action of guava leaf extract (GLE) at the molecular level in treating DM. A low dose of streptozotocin (STZ) was used to induce experimental diabetes in animals. Rats were treated with GLE at different concentrations (100, 200, and 400 mg/kg b.w.). The standard drug glibenclamide (GB) (600 *μ*g/kg b.w.) was used for comparison. The diabetic rats showed a reduced level of insulin, accompanied by exaggerated levels of blood glucose, lipid peroxidation product, and augmented expressions of inflammatory cytokines, and showed reduced levels of antioxidants compared to the control rats. Supplementation with GLE counteracted the consequences of STZ. It suppresses the oxidative stress and inhibits the state of inflammation and the results are almost similar to that of standard drug group (GB group 5). Our present research, therefore, provides useful data concerning guava leaf extract by a thorough assessment in diabetes management. Being a natural product, additional analysis on GLE can shed light on finding effective phytochemicals within the field of diabetes mellitus.

## 1. Introduction 

Diabetes mellitus (DM) is a group of metabolic diseases resulting in an increased blood glucose level (hyperglycemia). A prolonged hyperglycemia is the key indicator of the metabolic illness diabetes mellitus. Oxidative stress plays a vital role in the pathogenesis of DM [1]. An imbalance within the redox status or the production and detoxification of reactive oxygen species (ROS) end up in injury to varied tissues and therefore the condition is termed as oxidative stress. It is assessed by the extent of the reaction product of oxidative harm, DNA oxidation, lipid peroxidation, and protein oxidation [2]. In diabetes, oxidative stress is caused by both an increased formation of plasma free radicals and a diminution in antioxidant defenses. Hyperglycemia might enhance the production of free radicals and provoke oxidative stress which will also add to the redoubled risk for coronary artery illness in diabetes [3]. Therapeutic choices for treating diabetes embrace sulfonylureas and alternative hypoglycemic agent secretagogues, alpha-glucosidase inhibitors, biguanides, thiazolidinediones, and insulin.

Guava (*Psidium guajava* L.) is a 15 m height tree with numerous nutritional values. The nutritional value of guava fruit is known throughout the globe. Aside from this, many chemical and pharmaceutical companies use numerous parts of guava tree [4]. The guava leaf has gone through phytochemical analysis and found to have alkaloids, carotenoids anthocyanins, vitamin-C, and triterpenes [5–9]. The anti-inflammatory and analgesic effects of* P. guajava* leaves (70% ethanolic extract) were established to be effective in rats using the carrageen induced hind paw oedema model [10]. The guava leaves conjointly cure numerous other diseases [11]. A study states that to treat the inflammation of the kidney, the fresh guava leaves are used [12]. The pulped leaves are used for treating piles in Congo [13].

Utilization of folk herbal medication knowledge by autochthonal cultures is not solely useful for preserving their culture however conjointly useful for synthesizing new medicine. There's less systematic study on the effectiveness, let alone the mechanism of guava leaf in treating diabetes. Hence we tend to investigate the effectuality of GLE against DM and its associated hyperglycemia, oxidative stress, and inflammation (NF-kB regulation). The results from our study clearly indicate that GLE has the power to inhibit numerous pathological conditions related to DM. The study on the regulation of NF-kB by GLE evidences that inflammation plays a major role within the DM. In the future, studies on the role of GLE on insulin signaling pathway and it is interwoven with oxidative stress and inflammation might derive a conclusion on the therapeutic effects of GLE on DM.

## 2. Materials and Methods

### 2.1. List of Chemicals

Sigma-Aldrich (Shanghai, China) is the source for streptozotocin (STZ) and glibenclamide (GB). Primary antibodies for interleukin-6 (IL-6), nuclear factor-kappa B (NF-kB), and tumor necrosis factor-*α* (TNF-*α*) were purchased from Abcam (China). Secondary antibody goat-anti-rabbit (CW0156) was purchased from CW Biotech, China. All different analytical grade chemicals and reagents were purchased from Shanghai Yuanye Biotechnology Co. (Shanghai, China).

### 2.2. Collection and Sample Preparation of Guava Leaves

The guava leaves from the species* P. guajava *were collected from the plant gardens from southern China (Meizhou, Guangdong, China). Guava leaves were carefully separated and cut into small thin pieces and dried at room temperature for 2 days.

### 2.3. Preparation of Guava Leaf Extract (GLE)

The dried leaves were ground and changed into powder form. The desktop decoction extractor (YFT20, Beijing Donghuayuan Medical Equipment Co., Ltd., Beijing, China) was used to prepare the extract. In this process, 100 g of dried guava leaves was boiled in 1.5 L of water for about 4 hours. Further the extract was filtered using a Whatman No. 4 filter paper and dried using a rotary evaporator at 60°C. The dried extract was converted into a powder form which was utilized for the preparation of desired concentrations of the extracts. The extracts were stored at 4°C in sterile bottles until further use. While performing the experiments the powdered GLE was dissolved in water and administered to the animals.

### 2.4. Experimental Animals and Conditions

Healthy male Wistar rats (180–220 g) were procured from Southern Medical University (Guangzhou, China). The experiments were executed in accordance with the principles issued by the National Institute of Health (NIH) Guideline for the experimental animals care and use. The ethics committee (Animal) of Zhuhai Campus of Zunyi Medical University, China, issued the approval to hold out this experimental protocol that additionally conforms to the rules for ethical conduct within the animals use and care. Rats were placed in a polypropylene cage and in a temperature of 25 ± 2°C with relative humidity (45%  ±  5%) in 12 h light and 12 h dark condition. Before experiments, rats were placed within the animal house for the period of two weeks. The standard pellet diet (obtained from Southern Medical University, Guangzhou, China) was a balanced food composed of protein 21.1%, fat 5.1%, carbohydrates 60.0%, fiber 3.9%, vitamins 2.0%, and minerals 7.9%.

### 2.5. Induction of Experimental Diabetes in Animals

A freshly prepared 0.1 M, pH 4.5 citrate buffer was used to dissolve the STZ and maintained on ice prior to use. Animals were kept on overnight fasting to induce diabetes (low dose STZ model) by an intraperitoneal injection of STZ at a dose of 40 mg/kg b.w. The elevated plasma glucose was determined by Accu–Chek commercial kit (Roche diagnostics, Mannheim, Germany) and rats having fasting glucose greater than 250 mg/DL were screened and considered as diabetic rats and used for further studies.

### 2.6. Experimental Design of Animal Study

The animals were grouped into seven groups (*n* = 6), and a total of 42 rats (30 diabetic and 12 control) underwent the study. Treatment with GLE was started on the third day after STZ induction. GLE was dissolved in water and variant doses of GLE were administered using an intragastric tube orally for 45-day duration.

  Experimental group I: control rats.
  Experimental group II: GLE control (400 mg/kg b.w.).
  Experimental group III: diabetic rats.
  Experimental group IV: diabetic + GLE (100 mg/kg b.w.).
  Experimental group V: diabetic + GLE (200 mg/kg b.w.).
  Experimental group VI: diabetic + GLE (400 mg/kg b.w.).
  Experimental group VII: diabetic + GB (600 *μ*g/kg b.w.).

When the experimental period has come to an end, the animals were kept in the fasting condition whole night and then animals were anesthetized by an intramuscular injection of ketamine hydrochloride at a dose of 24 mg/kg b.w. and the animals were killed. For the estimation of insulin and glucose, blood samples were collected into tubes containing an anticoagulant. An ice cold saline was kept ready to wash the dissected tissues (liver, kidney, and pancreas) and later stored at −80°C for further experiments.

### 2.7. Quantification of Phenolics in Guava Leaf

To quantify the total phenolic and total flavonoids contents in guava leaves, the dry guava leaves were extracted with different solvents (ethanol/water (1 : 1, v/v), methanol/water (1 : 1, v/v), ethanol, water, and methanol), respectively.

#### 2.7.1. Determination of Total Phenolic Compounds

The method of Taga et al. [14] was used to confirm the total phenolic content in guava leaf extract (GLE) and expressed as equivalents of gallic acid. The acidified (3 g/L HCl) methanol/water (60 : 40 v/v) and 100 *μ*L of each were added separately to 2 mL of 2% Na_2_CO_3_ to prepare the samples and standards. Five minutes later 100 *μ*L of 50% Folin–Ciocalteu reagent was added to the samples and kept at RT for 30 min and employing a spectrophotometer absorbance was measured at 750 nm (Shimadzu, 160A). A blank has been prepared with all solvents, excluding samples and standards. The 10–100 *μ*g/mL concentration of gallic acid was prepared. The phenolic acid concentration obtained from guava leaf extract was compared with standards. A triplicate of analyses was carried out and calculated.

#### 2.7.2. Determination of Flavonoid Content Assay

The Moreno et al. method was employed to estimate the flavonoid content [15]. In the experimental procedure, the following chemicals were added: (1) 0.1 mL of 10% aluminum nitrate, (2) 0.1 mL of 1 mL/L aqueous potassium acetate, and (3)4.3 mL of 80% ethanol added along with 1 mL of different concentration of extracts. Absorbance (415 nm) was measured in a dark room after a 40 min incubation at RT. Quercetin was used as a standard for estimating flavonoids.

#### 2.7.3. Determination of Phenolic Acids Composition in Guava Leaves by High Performance Liquid Chromatography (HPLC)

The method of Luthria and Pastor–Corrales (2006) was used to extract the overall phenolic acids. A 50 mL of methanol/water/acidic/butylated hydroxytoluene at a ratio of 85 : 15 : 0.5 : 0.2 was used to extract the leaf sample (0.5 g). The content was kept in an orbital shaker for 3 h at RT at 300 revolutions per minute, followed by a centrifugation at 8000*g* for 20 min. To get rid of the surplus solvents the extracts were concentrated under vacuum at 45°C. 2 mL of 25% methanol was used to dissolve the dry residue. The methanol sample aliquot was filtered into HPLC sample container using a 0.2 mm filter to get rid of the impurities and insoluble substances present in it and therefore the sample was stored at 4°C for HPLC analysis.

HPLC phenolic acid analysis was performed in line with the method demonstrated by Xu and Chang [16]. For a water associated (Milford, MA, USA) chromatography system alongside a 720 model system controller, a model 7125 sample injector, and a 6000 model “A” solvent delivery system, the values are detected at 280 nm employing a model 418 LC uv detector. For separation at 40°C, a 4.6 × 250 mm, 5 *μ*m, Zorbax Stable bond analytical SB–C18 column (Agilent Technologies, Rising Sun, MD, USA) was used. The mobile phase comprised solvent “A” (0.1% TFA) and solvent “B” (methanol), at 0.7 mL/min of flow rate and 20 *μ*L of injection volume. The good separation was found through gradient elution and all peaks were identified, and phenolic acids were quantified with a relative retention time of external standards. The phenolic acids contents were expressed as *μ*g/mg on dry weight basis.

### 2.8. Biochemical Parameters

#### 2.8.1. Insulin and Glucose Estimation

Accu–Chek commercial kit was used to determine the blood glucose levels (Roche Diagnostics, Mannheim, Germany). A Merck Millipore commercial kit was used to analyze the levels of plasma insulin (Darmstadt, Germany).

#### 2.8.2. Estimation of Lipid Peroxidation (LPO) Markers

LPO markers in liver, kidney, and pancreas were estimated with UV/VIS spectrophotometer adopting the methods and procedures made by Fraga et al. [17] and Jiang et al. [18]. A 2 mL volume of thiobarbituric acid (TBA)–trichloroacetic acid (TCA)–HCl reagent (0.37% TBA, 0.25 M HCl, and TCA, 1 : 1 : 1 ratio) was mixed with 0.1 mL of tissue homogenate and warmed in an exceeding water bath for a period of 15 min and further it underwent centrifugation at a speed of 3500xg for about 10 min at 37°C, and later 535 nm was used to measure the intensity of the supernatant. Lipid hydroperoxide values were manifested as mM/100 g of tissue. To 0.9 mL of Fox reagent (88 mg of butylated hydroxyl toluene (BHT), 0.8 mg of ammonium iron sulfate, and 7.6 mg of xylenol orange) were added 90 mL of alcohol and 10 mL of 250 mm sulfuric acid. 0.1 mL of tissue homogenate was added and incubated at RT for 30 min and 560 nm was used to measure the absorbance.

#### 2.8.3. Determination of Catalase Activity (CAT)

Beers and Sizer [19] were used to measure the activity of catalase. The reaction mixture is the combination of 0.1 mL of tissue homogenate (1.5 mL), 1.0 mL of 0.01 M phosphate buffer (pH 7.0), and 0.4 mL of 2 M H_2_O_2_. The reaction was arrested by the addition of 2.0 mL of dichromate-acetic acid reagent (potassium dichromate (5%) and glacial acetic acid in a ratio of 1 : 3). 620 nm was used to read the absorbance; *μ*M of H_2_O_2_ consumed/min/mg protein is the unit used to denote CAT activity.

#### 2.8.4. Determination of Superoxide Dismutase Activity (SOD)

The Sun and Oberley [20] methodology was used to verify the activity of SOD. Using 1 mL of water the tissue homogenate was diluted. To this diluted homogenate were added a cooled 1.5 mL of chloroform and 2.5 mL of ethanol and then they were centrifuged. The supernatant was used to estimate the activity of the enzyme. The mixture of this assay is a composition of 1.2 mL of sodium pyrophosphate buffer (0.025 M, pH 8.3), 0.1 mL of 186 *μ*M potassium metabisulfite, 0.2 mL of 780 *μ*M NADH, 0.3 mL of 30 *μ*M nitroblue tetrazolium (NBT), befittingly diluted enzyme preparation, and water. The total volume was made in 3 mL. NADH was added as an initiation of the reaction. Followed by 90 min incubation at 30°C, one mL of glacial acetic acid was used to arrest the reaction. The mixture was jolted vigorously upon mixing with 4 mL of* n*-butanol. A 560 nm was used to measure the compound concentration and the specific activity of the enzyme was expressed as enzyme required for 50% inhibition of NBT reduction/min/mg protein.

#### 2.8.5. Determination of Glutathione Peroxidase Activity (GPx)

The Rotruck et al. [21] methodology was used to measure the activity of GPx. Briefly, the reaction mixture containing 0.2 mL tissues was homogenized in 0.2 mL of 0.4 M phosphate buffer (pH 7.0), 0.1 mL of 10 mM sodium azide, phosphate buffer, 0.1 mL of 0.2 mM H_2_O_2_, pH 7.0, and 0.2 mL glutathione. The incubation time was 10 min at 37°C and the reaction was arrested by the addition of 0.4 mL 100% TCA and subsequent centrifugation at 3200*g* for 20 min was done. The glutathione content of the supernatant was assayed using Ellman's reagent (19.8 mg 5, 50-dithiobisnitrobenzoic acid in 100 mL 0.1% sodium nitrate). The *μ*g of GSH consumed/min/mg protein is denoted as a unit to measure the activity of the enzyme.

#### 2.8.6. Determination of Glutathione Reductase Activity (GR)

The Horn and Burns [22] methodology was used to verify the GR activity. The reaction mixture is composed of 0.5 mL of GSSG, 1 mL of phosphate buffer, 0.2 mL of NADPH, and 0.5 mL of EDTA. The reaction mixture was made to the final volume by adding 3 mL of distilled water. A 340 nm was used to measure the absorbance after the addition of 0.1 mL of tissue homogenate. The activity of GR was measured as *μ*moles of NADPH oxidized/min/mg protein.

#### 2.8.7. Estimation of Reduced Glutathione (GSH)

The Beutler and Kelly [23] method was used to measure the concentration of GSH. A yellow derivative has been formed after the reaction between the supernatant with 5, 50-dithio-bis-2-nitrobenzoic acid (DTNB). The intensity was read at 412 nm.

#### 2.8.8. Determination of Vitamin-C (VIT-C)

The Omaye et al. [24] method was used to estimate VIT-C. A mixture is made of 1.5 mL of 6%, and TCA and 0.5 mL of tissue homogenate were centrifuged at 3500xg for 10 min. 0.5 milliliters of dithiobis-2-nitrobenzoic acid (DNPH) reagent was mixed with the above supernatant and incubated at 37°C for about 3 hours, followed by an addition of 2.5 mL of 85% sulfuric acid, and kept for incubation about 30 min. A 530 nm was used to measure the absorbance. A standard series of ascorbic acid was taken as 10–50 mg and compared with the samples. The *μ*M/mg tissue is denoted as the unit to measure the levels of ascorbic acid.

#### 2.8.9. Determination of Vitamin-E (VIT-E)

The Baker et al. [25] method was used to measure the activity of VIT-E. A reaction mixture was made by adding 1 mL of lipid extract with 1.5 mL of ethanol and 2 mL of petroleum ether, mixed. The resultant mixture was further centrifuged at a speed of 3000xg for 10 min. At 80°C the supernatant was evaporated to become dry, and then to the dry sample was added 0.2 mL of ferric chloride solution and of 2-1-dipyridyl solution each. The mixture was kept in the dark for 5 min, followed by addition of 2 mL of butanol. 520 nm was used to measure the ultimate absorbance. A standard *α*-tocopherol in a range of 10–100 mg was compared with the results of samples. The *μ*M/mg of tissue is denoted as the unit to measure the activity of VIT-E.

#### 2.8.10. Estimation of Protein

The total protein was estimated by the method of Lowry et al. [26]. An aliquot of cell lysate and tissue homogenate was diluted to 1.0 mL with saline, then 1.0 mL 10% TCA was added. The resulting mixture was centrifuged, supernatant was discarded, and using 1.0 mL of 0.5 N NaOH the precipitate was dissolved. Aliquots were taken for the estimation from the above said step. A 4.5 mL alkaline copper reagent was added and the contents were incubated at 37°C for 10 min, followed by the addition of Folin–Ciocalteu reagent. A series of standard solution in a range of 20–100 *μ*g and a blank were processed in the same way. The intensity of the blue color developed was read at 620 nm after 20 min. The protein values are expressed as mg/g tissue.

### 2.9. Expression of Inflammatory Cytokines

RIPA lysis buffer was used to homogenize the pancreas. Then, the homogenate was placed on cold condition for 30 min, followed by centrifugation at 4°C, the centrifuge was precooled before beginning the experiment, and a speed of 20,000*g* was used for 20 min and also the supernatant obtained after the centrifugation was used as a sample and pellets were thrown. Samples containing 50 *μ*g of total protein were loaded on a SDS polyacrylamide gel and then separated by electrophoresis. The SDS-PAGE gel was then transferred onto a PVDF membrane (Millipore) followed by electrophoresis. A block buffer containing 5-hitter nonfat dry milk powder or 5-hitter BSA was used to incubate the membrane for 2 h. This was done to cut back the nonspecific binding areas and then incubated in NF-kB-p65 monoclonal, TNF-*α*, and IL-6 (rabbit monoclonal; 1 : 250) and*β*-actin (rabbit monoclonal; 1 : 1000) in BSA throughout the night at 4°C. Next day the membranes were treated with their corresponding secondary antibodies (anti-rabbit igg conjugated to horseradish peroxidase) for 2 h at normal room conditions. TBST buffer was used to wash the blot membrane and the washing was carried out for 30 min. Chemiluminescence protocol was used to visualize the immune-reactive protein (GenScript ECL kit, Piscataway, NJ, USA, and Image Quant LAS 500) to review the densitometric analysis of the respective protein bands within the gel and a gel image study program was used. A standard protein*β*-actin was used to compare the bands with other proteins.

### 2.10. Histopathological Study

The tissues to be examined (liver, kidney, and pancreas) are fixed in 10% normal saline for 48 h and for dehydration a different mixture of water and ethyl alcohol was used and xylene was used to clean the slides and paraffin wax was used to fix the tissue sections on slides. A 4-5 *μ*m of liver, kidney, and pancreas sections was made and then stained with hematoxylin and eosin (H & E) dye, the stained slides were mounted with a neutral deparaffinated xylene medium, and the visualization of slides was done using a light microscope at 40x.

### 2.11. Statistical Analysis of Variance

All data were presented as the mean ± standard deviation (SD) of experiment numbers (*n* = 6). One-way analysis of variance (ANOVA) using SPSS Version 15 (SPSS, Cary, NC, USA) was used to determine the statistical significance and variance and Duncan's multiple range test (DMRT) was used to determine the individual comparisons. When *p* < 0.05, values are considered statistically significant.

## 3. Results 

### 3.1. Total Phenolic and Flavonoid Contents in Guava Leaf Extracts by Different Solvent

**Figure 1:**
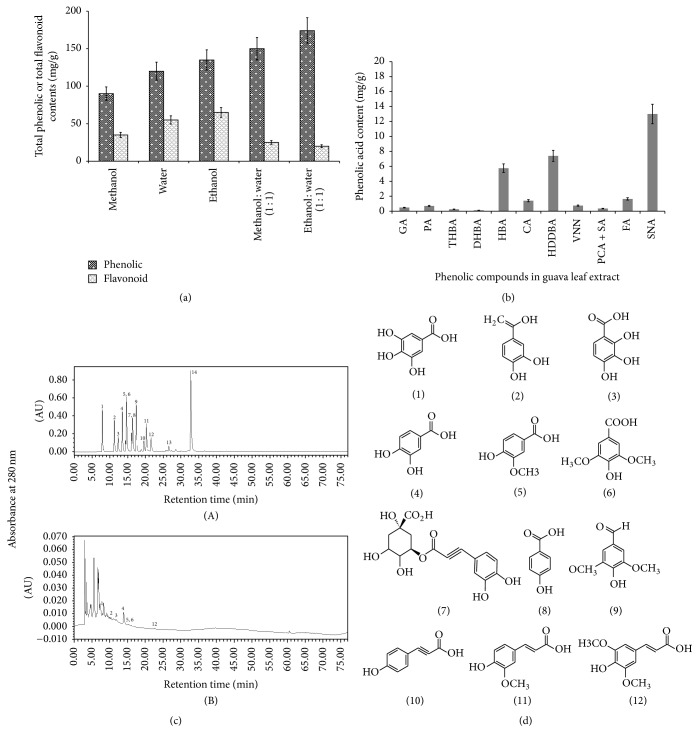
(a) Total phenolic and flavonoid contents of guava leaf (dry weight basis) by using different solvent systems. (b) Quantification of phenolic compounds in guava leaf extracts by high performance liquid chromatography (HPLC). GA, gallic acid; PA, protocatechuic acid, THBA, 2,3,4-trihydroxybenzoic acid; DHBA, 3,4-dihydroxybenzaldehyde; HBA, 4-hydroxy benzoic acid; CA, chlorogenic acid; HDDBA, 4-hydroxy-3,5-dimethoxybenzoic acid; VNN, vanillin; PCA + SA,* p*-coumaric acid plus syringaldehyde; FA, ferulic acid; and SNA, sinapic acid. (c) HPLC chromatographs of phenolic compound standards (A) and guava leaf extracts (B): (1) gallic acid (GA), (2) protocatechuic acid (PA), (3) 2,3,4-trihydroxybenzoic acid (THBA), (4) 3,4-dihydroxybenzaldehyde (DHBA), (5) 4-hydroxybenzoic acid (HBA), (6) gentisic acid, (7) chlorogenic acid (CA), (8) vanillic acid (VA) plus caffeic acid (CAA), (9) 4-hydroxy-3,5-dimethoxybenzoic acid (HDDBA), (10) vanillin (VNN), (11)* p*-coumaric acid (PCA) + syringaldehyde (SA), (12) ferulic acid (FA), (13) sinapic acid (SNA), and (14) salicylic acid (SAL). (d) Possible phenolic compounds identified in guava leaf extract. (1) Gallic acid (GA), (2) protocatechuic acid (PA), (3) 2,3,4-trihydroxybenzoic acid (THBA); (4) 3,4-dihydroxybenzaldehyde (DHBA), (5) 4-hydroxybenzoic acid (HBA), (6) gentisic acid, chlorogenic acid (CA), (7) 4-hydroxy-3,5-dimethoxybenzoic acid (HDDBA), (8) vanillin (VNN), (9) and (10)* p*-coumaric acid (PCA) + syringaldehyde (SA), (11) ferulic acid (FA), and (12) sinapic acid (SNA).

The variant of solvent extract obtained from guava leaf was tested for its total phenolic and flavonoid content and the results were shown in Figure 1(a). The yield of guava leaf extracts (GLE) was found to be 5.5%, 5.1%, 4.6%, 4.4%, and 4.1% in various solvent extracts such as ethanol/water (1 : 1, v/v), methanol/water (1 : 1, v/v), ethanol, water, and methanol, respectively. The yield of GLEs was calculated on the dry weight basis. Maximum total phenolic content (174.56 mg/g on dry weight basis) was achieved in the ethanol/water (1 : 1, v/v) extract, while the maximum total flavonoid content (65.12 mg/g on dry weight basis) was found in ethanol alone extract. Of all the solvents used, total phenolic contents were found to be lower in methanol/water (1 : 1) extract followed by ethanol, water, and methanol alone extracts. The flavonoid contents were found to be lower in water followed by methanol, methanol/water (1 : 1), and ethanol/water (1 : 1) extracts. Previous studies report that guava leaf contains terpenoids [5, 6], flavonoids [27, 28], and tannins [29].

### 3.2. Identification and Quantification of Phenolic Compounds in Guava Leaf Extract by HPLC

The Folin–Ciocalteu method gives total phenolic contents in the guava leaf extracts since the reactivity is different for different polyphenolics so the determination of phenolic compounds is not specific, whereas HPLC analysis offers more exact information about individual compounds. The phenolic acids are generally read at 280 nm. Standards were used to compare the results by comparing the retention times and UV spectra of samples. The phenolic compounds in the guava leaf extracts are identified and quantified by comparison with authentic standards and the quantification is expressed as mg/g weight of the extract. The quantification of phenolic compounds in the guava leaf extract is presented in Figure 1(b) and a typical HPLC chromatogram was shown in Figure 1(c). Fourteen phenolic compounds such as gallic acid, 3,4-dihydroxybenzoic acid, 2,3,4-trihydroxybenzoic acid, 3,4-dihydroxybenzaldehyde, caffeic acid, gentisic acid, chlorogenic acid, 4-hydroxybenzoic acid, vanillic and syringic acid, vanillin,* p*-coumaric acid plus syringaldehyde, ferulic acid, sinapic acid, and salicylic acid were analyzed, and thirteen compounds were detected in guava leaf extracts. Among 11 detected phenolic acids, sinapic acid (SNA, 13 mg/g) was found predominant phenolic compound in guava leaf extracts followed by 4-hydroxy-3,5-dimethoxybenzoic acid (HDDBA, 7.4 mg/g); 4-hydroxy benzoic acid (HBA, 5.75 mg/g); ferulic acid (FA, 1.64 mg/g); chlorogenic acid (CA, 1.4 mg/g); vanillin (VNN, 0.74 mg/g); protocatechuic acid (PA, 0.7 mg/g); gallic acid (GA, 0.48 mg/g);* p*-coumaric acid plus syringaldehyde (PCA + SA, 0.35 mg/g); 2,3,4-trihydroxybenzoic acid (THBA, 0.26 mg/g); and DHBA, 3,4-dihydroxybenzaldehyde (DHBA, 0.12 mg/g). Previously, 3,4-dihydroxybenzoic acid, gallic acid, chlorogenic acid, 4-hydroxybenzoic acid, syringic acid, caffeic acid, catechin, 2-hydroxybenzoic acid, ferulic acid, epicatechin, naringin, morin, and quercetin were reported in guava leaf extracts by various researchers [28, 30–34].

### 3.3. Influence of GLE on Insulin and Glucose

**Figure 2:**
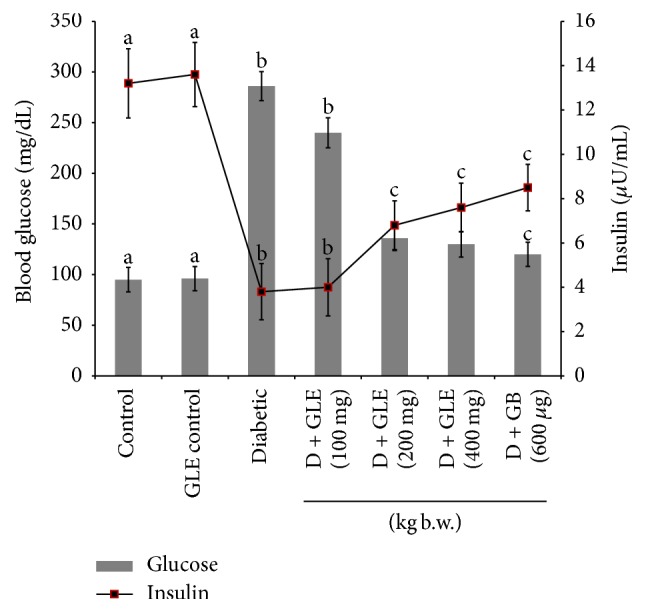
Effect of GLE on plasma glucose and insulin levels. Each value is mean ± SD of 6 rats in each group. In each bar, means with different superscript letters (a, b, and c) differ significantly at *p* < 0.05 (DMRT). D: diabetic and GLE: guava leaf extract.

Figure 2 elucidates the hyperglycemic condition diabetic rats, whereas, on supplementation with GLE different doses at 100, 200, and 400 mg/kg b.w., the plasma glucose level was significantly (*p *< 0.05) reduced. Inversely the concentration of insulin (plasma) was declined in the diabetic rats, and upon supplementation, with GLE the plasma insulin was significantly (*p* < 0.05) increased near to normal. The efficient results were seen in group 5 (200 mg/kg b.w.) so the remaining experiments were carried out using 200 mg/kg b.w. as an effective dose. The diabetic rats treated with GB showed similar results as GLE treatment group with not much variance. Hence the results of GLE can be compared with that of standard drug GB.

### 3.4. Influence of GLE on Lipid Peroxidation Markers

The levels of lipid peroxidation markers were elevated in the diabetic rats (Table 1), whereas, upon GLE supplementation (group 4), the levels were reduced markedly (*p* < 0.05), in comparison to the diabetic group (group 3). The similar results were obtained in the GB treated rats. The results of GLE and GB are not statistically significant with each other.

### 3.5. Influence of GLE on Enzymatic Antioxidants

Table 2 shows that the concentrations of antioxidative enzymes (SOD, CAT, GPx, and GR) were decreased in diabetic rats, whereas, on GLE supplementation, the levels of these enzymes were increased significantly (*p* < 0.05) as compared to the nontreated diabetic group (group 3). The similar results were obtained in the GB treated rats. The results of GLE and GB are not statistically significant with each other.

### 3.6. Influence of GLE on Nonenzymatic Antioxidants

Table 3 shows that the concentrations of Vit-E, Vit-C, and GSH were decreased in the diabetic rats, whereas, on GLE supplementation, these cellular antioxidants were increased significantly (*p* < 0.05). The similar results were obtained in the GB treated rats. The results of GLE and GB are not statistically significant with each other.

### 3.7. Influence of GLE on the Liver, Kidney, and Pancreas Histology

**Figure 3:**
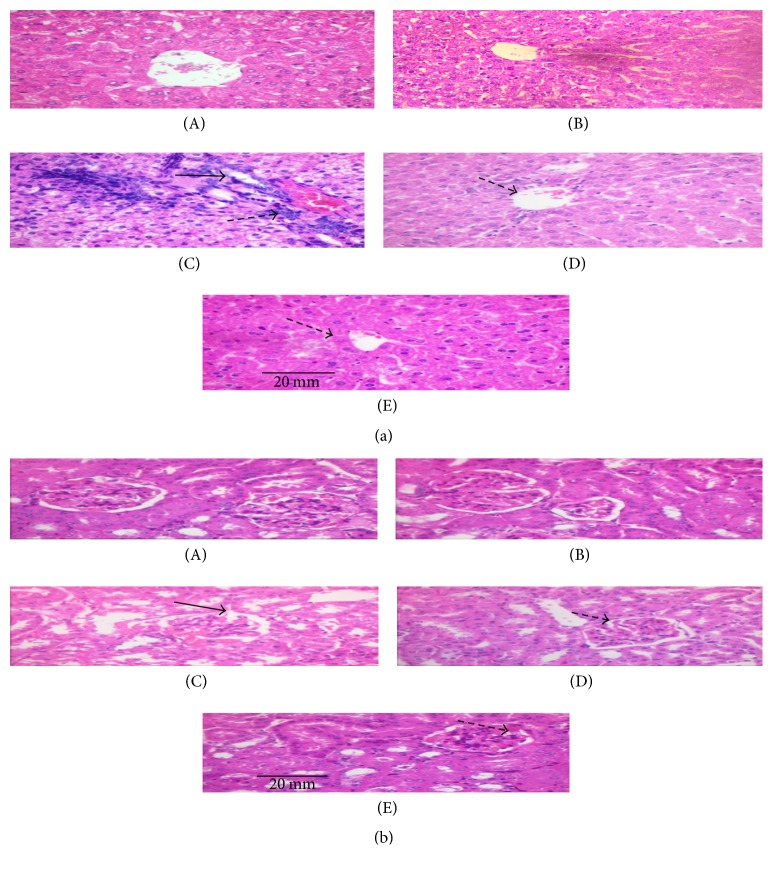
(a) Effect of GLE on the liver histology of the control and experimental rats (A) and (B) shows normal histology of liver with central vein. (C) Liver histology of diabetic rats (group 3) shows congestion of sinusoidal dilatation (indicated by arrow →), inflammation of the central vein (indicated by arrow *⇢*), and focal necrosis in the hepatocytes in diabetic control rats. (D) Liver histology of treatment group (group 4) shows reduced inflammation, a significant reduction in sinusoidal dilatation (indicated by arrow *⇢*), and tissue architecture near to that of normal. (E) Liver histology of standard drug group (group 5) also shows reduced inflammation, a significant reduction in sinusoidal dilatation (indicated by arrow *⇢*), and tissue architecture near to that of normal. (b) Effect of GLE on the kidney histology of the control and experimental rats (A) and (B) shows normal kidney histology of control (group 1) and GLE control (group 2). (C) Kidney histology of diabetic rats (group 3) showed multiple foci of hemorrhage, necrosis, and swelling of tubules (indicated by arrow →). (D) Kidney histology of treatment group (group 4) shows reduced necrosis and no swelling of tubules (indicated by arrow *⇢*). (E) Kidney histology of standard drug group (group 5) also shows reduced necrosis and no swelling of tubules (indicated by arrow *⇢*).

**Figure 4:**
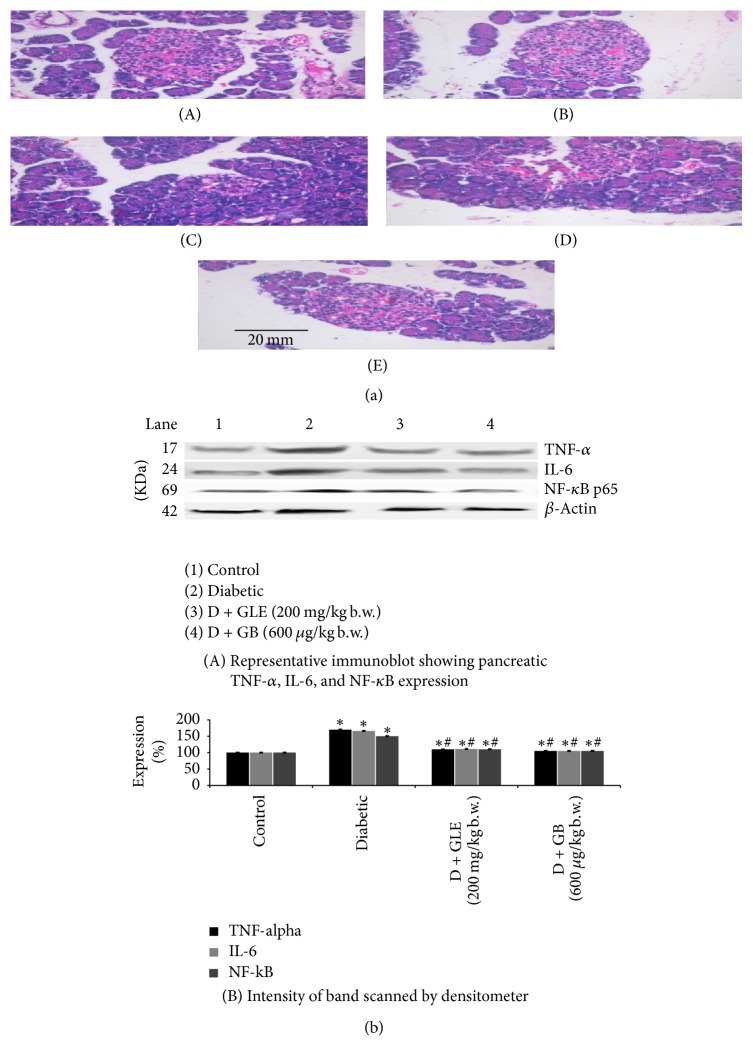
(a) Effect of GLE on the pancreas histology of the control and experimental rats.** (**A) and (B) show normal pancreas histology of control (group 1) and GLE control (group 2), (C) pancreas histology of diabetic rats (group 3) showed infiltration and destroyed islet cells of pancreas as a result of which they were significantly reduced in size and number, (D) pancreas histology of treatment group (group 4) shows well-granulated and prominent hyperplasticity of islets, and (E) pancreas histology of GB group (group 5) shows well-granulated and prominent hyperplasticity of islets. (b) Immunoblot of NF-kB, TNF-*α*, and IL-6 protein samples (50 *μ*g/lane) resolved on SDS-PAGE was probed with corresponding antibodies. Each lane was analyzed by densitometry and the expression in the control was considered as 100%. The column heights are the means ± SD of six determinants. ^*∗*^Significantly (*p* < 0.05) different from control groups and ^#^significantly different from STZ alone treated groups (*p* < 0.05).

Figures 3(a), 3(b), and 4(a) elucidate the pathological changes that occurred as a result of STZ in the liver, kidney, and pancreas, which could exhibit the ability of GLE in protecting the tissue from damage in STZ induced diabetic rats. Liver of diabetic rats shows focal necrosis, inflammation of the central vein, and the congestion of sinusoidal dilatation in the hepatocytes of diabetic rats. After six weeks of treatment with GLE, the liver tissues from the D + GLE group show liver cells arranged neatly and no distinct inflammation of central vein was seen. A similar kind of changes was observed in the GB group. GLE offers a vital protection against STZ induced damage. Kidney of diabetic rats showed necrosis, swelling of tubules, and multiple foci of hemorrhage. After six weeks of treatment with GLE, the kidney tissues from the D + GLE group show renal tubular structures in good condition, no necrosis, and swelling of tubules compared with the diabetic group. Glibenclamide supplemented group showed a similar improvement. No changes in tissue architecture were observed in hepatic and renal tissues of control rats. The histopathological study of the pancreas shows. In Figure 4(a), in (A) and (B) control group and control along with GLE show normal pancreatic islet. In (C), STZ induced diabetic rats show infiltration of fats and damaged islet cells of pancreas as a result of which they were significantly reduced in size and number. In (D), diabetic + GLE (200 mg/kg b.w.) treated rats show islets with proper granules and also show the cell hyperplasty. In (E), the similar results as group 4 GLE treatment rats were obtained in the GB treated rats (Group 5). This evidences that GLE have the ability to inhibit the effects of STZ on various tissues comparable to that of GB standard drug.

### 3.8. Influence of GLE on Inflammatory Markers

Figure 4(b) shows the immunoblot quantifying interleukin-6 (IL-6), tumor necrosis factor (TNF-*α*), and nuclear factor-kappa B (NF-*κ*B) expression. Control group rats show normal protein expression and diabetic rats (group 2) show increased expression of NF-*κ*B, TNF-*α*, and IL-6. Supplementation with GLE to diabetic rats (group 3) significantly downregulated the expression of inflammatory cytokines (NF-*κ*B, TNF-*α*, and IL-6). Diabetic rats supplemented with GB showed similar results as the GLE treatment group which could prove the ability of GLE in inhibiting inflammation compared to a standard drug. The *β*-actin was used as the internal standard.

## 4. Discussion 

Diabetes mellitus (DM) is a combination of heterogeneous disarray commonly presenting with the incidence of hyperglycemia and glucose intolerance, as a result of lack of insulin, defective insulin action, or both [35]. There is enough proof to demonstrate that hyperglycemia plays an important role in initiating oxidative stress and different complications associated with DM. In the current study, we have examined the role of bioactive compounds in GLE such as phenolic compounds on antidiabetic activity in STZ evoked diabetic rat model system. There are several reasons for choosing GLE; the important aspect we considered is that it has no side effects and no toxicity even at higher doses. In an interesting study by Kobayashi et al. [36], the results suggest that the oral administration of guava leaf extract at two different concentrations of 200 and 2000 mg/kg/day caused no abnormal toxic effects in rats which indicates that guava leaf extract has no side effects even at very high doses. Another interesting study has revealed that guava leaf extract did not induce chromosomal aberrations; hence it proved that it does not exhibit any genotoxic effects at a high dose of 2000 mg/kg [37]. We determined the overall phenolic and total flavonoid content in the guava leaf (Figure 1(a)). Further, the phenolic compounds in the guava leaf were quantified and identified by high performance liquid chromatography (Figures 1(b), 1(c), and 1(d)). The foremost phenolic compound was found to be sinapic acid followed by different phenolic compounds in the extract. Phenolic compounds in guava leaf such as gallic acid, chlorogenic acid, 3,4-dihydroxybenzoic acid, 4-hydroxybenzoic acid, syringic acid, caffeic acid, catechin, 2-hydroxybenzoic acid, ferulic acid, epicatechin, naringin, morin, and quercetin were reported by numerous researchers [28, 30–34]. The major phenolic compound sinapic acid undergoes absorption and metabolism and was excreted in the urine as 3-hydroxy-5-methoxyphenylpropionic acid, dihydrosinapic acid, 3-hydroxy-5-methoxycinnamic acid, and unchanged sinapic acid. Previous reports have mentioned that the phenolic compounds in GLE showed a possible biological activity in in vitro and in vivo model systems [4, 30, 33, 38]. In the method of eliminating free radicals, GLE plays a significant role [30]. STZ, a chemical that causes toxicity specifically in the insulin synthesizing *β*-cell (Islet of Langerhans) of the pancreas in mammals, is employed oftentimes to develop a typical diabetic model [39]. The mechanism of action of STZ takes place by excess production of ROS that induces cytotoxicity in beta-cells of the pancreas, followed by reduced insulin production. Type 1 diabetes with partial destruction of the pancreas was evoked by a single STZ injection. We have chosen a low dose of STZ to induce mild type 1 DM.

Free radicals play a serious role in the onset and progression of late diabetic complication. This action could also be owing to its ability to wreck numerous components of the cell such as proteins, lipids, and DNA [40]. Results of our study found that the amount of glucose was inflated considerably within the diabetic group and reciprocally the levels of insulin were diminished; this condition was attenuated with treatment with GLE (200 mg/kg b.w.) (Figure 2). The levels of lipid peroxidation markers, inflated in diabetic rats, were considerably prevented within the treatment group (Table 1). With this context, Soman et al. [41] verified that GLE can act as good antioxidant by reducing the blood glucose. The standard drug group shows reduced glucose levels by its action on calcium channels of pancreas for the release of insulin which reverses the hyperglycemic condition.

The formation of O_2_ and its removal were kept under check in unstressed conditions. However, beneath the severe oxidative stress attack can overwhelm the production of O_2_. Hence, antioxidant enzymes create the foremost defense against ROS playing a major role in eliminating the toxic incomplete oxidation's toxic intermediates. The superoxide dismutase (SOD) is a major antioxidant enzyme involved in direct ROS elimination and superoxide radical made within the cells which are further converted into H_2_O_2_ and later on eliminated as H_2_O and singlet oxygen [42]. Catalase (CAT) (EC 1.11.1.6) is present predominantly within the peroxisomes, which quickly convert toxic H_2_O_2_ into H_2_O [43]. CAT is modified along with glutathione peroxidase (GPx) and antioxidant enzyme contains selenium [44]. The level of lipid hydroperoxides (LOOH) was reduced by antioxidant enzyme GPx in the presence of glutathione. NADPH-dependent reduction of oxidized glutathione (GSSG) to reduced glutathione (GSH) was catalyzed by glutathione reductase (GR) (EC 1.8.1.7). GR plays a significant role in upholding the adequate levels of reduced GSH through GSH redox cycle. The levels of antioxidants enzymes were seen increased in the group supplemented with GB and the results are compared with the GLE and found statistically not significant.

The nonenzymatic antioxidants, like *α*-tocopherol (Vit-E), ascorbic acid (Vit-C), and glutathione play a vital role as antioxidants. They are interconnected by utilization processes and have very important responsibilities, against lipid peroxidation [45]. The Vit-E and Vit-C participate in a vital task in oxidative stress by defending the cells from deleterious effects. Vit- E is a potent antioxidant, helps in detoxifying superoxide and H_2_O_2_ free radicals, and offers stability to the membranes [46]. Vit-C or ascorbate is often referred to as a water soluble vitamin. A reactive and presumably detrimental radical will intermingle with ascorbate. This reaction forms an ascorbate radical with least effects to the membrane. GSH is an intracellular antioxidant produced by glutathione reductase helping in scavenging the free radicals. Under severe stress condition, this system was unable to maintain the reaction status within the cell. In our study, the rats provided GLE (200 mg/kg b.w.) markedly inhibit the decline of those antioxidants and forestall the tissues from the deleterious effects of oxidative stress (Tables 2 and 3). In support of the above findings, Suganya et al. [47] found that GLE exhibits strong free radical scavenging effects and this perhaps is a key factor in combating oxidative stress. The antioxidant potential of GLE should be mainly due to the presence of phenolic acids, in particular sinapic acid. Sinapic acid is proven to possess strong antioxidant activity that 0.2 *μ*M of sinapic acid can inhibit 33.2% of DPPH radical [48].

Even though the studies of varied antioxidant molecules are established to be vital, analyzing the histologic changes holds vital importance in explaining the disease mechanism. Histopathological findings of diabetic rats displayed the central vein inflammation, sinusoidal dilatation, and hepatocyte focal necrosis (Figure 3(a)). Multiple foci of hemorrhage, swelling of tubules, and necrosis were determined within the kidney (Figure 3(b)). STZ induced diabetic rats show fatty infiltration and destroyed islet cells of pancreas as a result of which they were considerably reduced in size and number (Figure 4(a)). The damage urged that the conventional detoxification process was impaired. The abovesaid alterations were reduced significantly in diabetic rats treated with GLE. Thus, histopathological observations conjointly support the concept that GLE reduces the burden of oxidative stress and protects the hepatic, renal, and pancreatic tissues in diabetic rats.

The various transcription factors like NF-*κ*B, PPAR-*γ*, p53, Nrf2, AP-1,*β*-catenin/Wnt, and HIF-1*α* can be activated by oxidative stress [49]. The activation of those transcription factors results in about 500-gene expression. With the assistance of those genes, these factors control numerous vital aspects like cell cycle regulatory molecules, growth factors, inflammatory cytokines, chemokines, and anti-inflammatory. Embryonic development, inflammation, tissue injury, and repair are controlled by an inducible transcription factor NF-kB [50]. NF-kB is present in inactive form alongside IkB in the cytoplasm beneath unstressed conditions. When cells are aroused by cytokines such as TNF-*α*, bounded IkB degrades, which results in the unmasking of NF-kB and further permits it to enter inside the nucleus for action. The transcription of NF-kB target inflammatory genes is initiated once NF-kB binds to the DNA. In our study administration of STZ results in hyperglycemia and severe oxidative stress, followed by inflammation, that was evidenced by the changes in the cytokines like IL-6, NF-kB, and TNF-*α* related to inflammation. Upon treatment with GLE, the rats showed improvement in their inflammatory expression (Figure 4(b)). The anti-inflammatory action of flavonoids is mainly due to its ability to inhibit the formation of proinflammatory mediators (e.g., adhesion molecules, cytokines, eicosanoids, and C-reactive protein) [38]. Phytochemical analysis of GLE shows a high content of flavonoids alongside alternative phytoconstituents, which can be responsible for its antihyperglycemic, antioxidative, and anti-inflammatory properties [51]. Glibenclamide is shown to inhibit inflammation from our results, which was in context with the findings of York et al., that GB can reduce the proinflammatory cytokine IL-6.

Results from our findings suggest that GLE has the great ability to cut back plasma glucose and oxidative stress and conjointly ameliorates the burden of inflammation in STZ evoked diabetic rats as confirmation by reduced glucose and restored antioxidant levels, besides reduced expression of inflammatory proteins. The beneficial effects of GLE on oxidative stress and inflammation in diabetic rats were well visualized in our histological studies. The mechanism of action of GLE may be owing to its ability to suppress the hyperglycemia by regulating the secretion of insulin from the pancreatic beta-cells and in turns it may ameliorate the oxidative stress and confirm the availability of enough antioxidant enzymes and it is well known that presence of oxidative stress and inflammation will initiate NF-kB activation; thus GLE alleviated the symptoms of diabetes by a successive regulation of hyperglycemia followed by oxidative stress and NF-kB pathway regulation. The regulating mechanism of GLE is mostly comparable to that of GB which was evidenced from the results of our study. Though various reports claim the antidiabetic potential of GLE, our research findings interrelated the main symptoms of diabetes such as hyperglycemia, oxidative stress, and inflammation scientifically and it enhances the scientific importance of this study and additional studies on its effectuality in clinical trials can add additional strength within the field of diabetes management in search of novel medicine with no side effects.

## Phytocompounds Analyzed in This Article

  3,4-Dihydroxybenzaldehyde and its PubChem CID is 70949.
  Chlorogenic acid and its PubChem CID is 1794427.
  Protocatechuic acid and its PubChem CID is 72.
  Gallic acid and its PubChem CID is 370.
  Vanillic acid and its PubChem CID is 8468.
  Caffeic acid and its PubChem CID is 689043.
  Vanillin and its PubChem CID is 1183.
 
*p*-Coumaric acid and its PubChem CID is 637542.
  Syringaldehyde and its PubChem CID is 8655.
  Ferulic acid and its PubChem CID is 445858.
  Sinapic acid and its PubChem CID is 637775.
  Salicylic acid and its PubChem CID is 338.

## Figures and Tables

**Table 1:** Tissue lipid peroxidation markers of the control and experimental rats.

Groups	Control	GLE control	Diabetic	D + GLE (200 mg/kg b.w.)	D + GB (600 *μ*g/kg b.w.)

TBARS (mmoles/mg tissue)					
Liver	0.67 ± 0.04^a^	0.66 ± 0.04^a^	2.86 ± 0.26^b^	0.99 ± 0.06^c^	0.91 ± 0.04^*c*^
Kidney	1.65 ± 0.07^a^	1.71 ± 0.06^a^	3.36 ± 0.34^b^	2.01 ± 0.08^c^	1.94 ± 0.06^*c*^
Pancreas	28.29 ± 5.45^a^	29.37 ± 4.87^a^	41.27 ± 5.21^b^	34.21 ± 4.19^c^	33.29 ± 4.32^*c*^

LOOH (mmoles/mg tissue)					
Liver	85.38 ± 7.89^a^	86.04 ± 7.35^a^	110.31 ± 8.29^b^	94.39 ± 7.39^c^	92.76 ± 7.11^c^
Kidney	71.28 ± 6.75^a^	70.37 ± 6.28^a^	120.38 ± 8.38^b^	85.63 ± 6.34^c^	82.38 ± 6.29^c^
Pancreas	33.89 ± 7.28^a^	32.89 ± 6.79^a^	69.11 ± 7.16^b^	45.39 ± 6.99^c^	41.98 ± 6.75^c^

CD (mmoles/mg tissue)					
Liver	65.84 ± 3.92^a^	66.43 ± 3.47^a^	81.29 ± 4.43^b^	69.28 ± 3.20^c^	64.98 ± 3.21^c^
Kidney	41.72 ± 2.06^a^	42.76 ± 2.11^a^	76.39 ± 3.86^b^	48.37 ± 2.87^c^	47.33 ± 3.01^c^
Pancreas	4.78 ± 0.28^a^	4.81 ± 0.32^a^	9.32 ± 0.53^b^	5.49 ± 0.26^c^	5.21 ± 0.22^c^

GLE: guava leaf extract. Values are given as means ± SD for six rats in each group. ^a^Group (group 2) with no significant difference compared to control group. ^b^Significantly different from control group at *p* < 0.05. ^c^Significantly different from diabetic group at *p* < 0.05. Duncan's Multiple Range Test (DMRT).

**Table 2:** Tissue enzymatic antioxidant status of the control and experimental rats.

Groups	Control	GLE control	Diabetic	D + GLE (200 mg/kg b.w.)	D + GB (600 *μ*g/kg b.w.)
SOD (50% NBT reduction/min/mg protein)					
Liver	7.67 ± 0.67^a^	7.81 ± 0.27^a^	3.86 ± 0.25^b^	5.32 ± 0.33^c^	5.67 ± 0.35^c^
Kidney	7.27 ± 0.55^a^	7.13 ± 0.45^a^	3.89 ± 0.65^b^	5.49 ± 0.27^c^	5.83 ± 0.29^c^
Pancreas	5.89 ± 0.48^a^	5.95 ± 0.53^a^	2.43 ± 0.42^b^	4.13 ± 0.56^c^	4.64 ± 0.58^c^

CAT (*μ*moles of H_2_O_2_ utilized/min/mg protein)					
Liver	84.81 ± 5.29^a^	83.29 ± 4.97^a^	57.28 ± 5.11^b^	71.29 ± 5.33^c^	73.45 ± 5.47^c^
Kidney	41.78 ± 3.98^a^	42.38 ± 3.26^a^	26.39 ± 2.54^b^	32.39 ± 3.21^c^	34.85 ± 3.33^c^
Pancreas	21.87 ± 2.11^a^	22.04 ± 2.41^a^	7.88 ± 1.25^b^	16.28 ± 1.78^c^	16.89 ± 1.68^c^

GPx (*μ*moles of GSH utilized/min/mg protein)					
Liver	9.01 ± 0.87^a^	8.99 ± 0.74^a^	4.87 ± 0.45^b^	8.11 ± 0.78^c^	8.27 ± 0.72^c^
Kidney	8.12 ± 0.69^a^	8.34 ± 0.45^a^	4.91 ± 0.39^b^	7.54 ± 0.81^c^	7.84 ± 0.83^c^
Pancreas	8.27 ± 0.75^a^	8.43 ± 0.78^a^	4.38 ± 0.37^b^	7.11 ± 0.57^c^	7.43 ± 0.58^c^

GR (*μ*moles of NADPH oxidized/min/mg protein)					
Liver	0.72 ± 0.06^a^	0.71 ± 0.06^a^	0.42 ± 0.02^b^	0.61 ± 0.06^c^	0.63 ± 0.06^c^
Kidney	0.62 ± 0.04^a^	0.63 ± 0.05^a^	0.34 ± 0.03^b^	0.50 ± 0.05^c^	0.54 ± 0.07^c^
Pancreas	0.71 ± 0.05^a^	0.70 ± 0.04^a^	0.32 ± 0.02^b^	0.59 ± 0.04^c^	0.65 ± 0.04^c^

GLE: guava leaf extract. Values are given as means ± SD for six rats in each group. ^a^Group (group 2) with no significant difference compared to control group. ^b^Significantly different from control group at *p* < 0.05. ^c^Significantly different from diabetic group at *p* < 0.05. Duncan's Multiple Range Test (DMRT).

**Table 3:** Tissue nonenzymatic antioxidant status of the control and experimental rats.

Groups	Control	GLE control	Diabetic	D + GLE (200 mg/kg b.w.)	D + GB (600 *μ*g/kg b.w.)
Vitamin C (mg/dL)					
Liver	1.45 ± 0.13^a^	1.43 ± 0.12^a^	0.77 ± 0.11^b^	1.30 ± 0.08^c^	1.33 ± 0.07^c^
Kidney	1.21 ± 0.11^a^	1.25 ± 0.13^a^	0.68 ± 0.09^b^	1.07 ± 0.12^c^	1.13 ± 0.13^c^
Plasma	1.57 ± 0.12^a^	1.60 ± 0.13^a^	1.17 ± 0.11^b^	1.38 ± 0.11^c^	1.48 ± 0.13^c^

Vitamin E (mg/dL)					
Liver	0.83 ± 0.08^a^	0.87 ± 0.05^a^	0.42 ± 0.03^b^	0.77 ± 0.06^c^	0.76 ± 0.07^c^
Kidney	0.67 ± 0.04^a^	0.68 ± 0.02^a^	0.33 ± 0.04^b^	0.54 ± 0.04^c^	0.62 ± 0.05^c^
Plasma	1.26 ± 0.08^a^	1.28 ± 0.08^a^	0.63 ± 0.05^b^	0.93 ± 0.06^c^	0.94 ± 0.05^c^

GSH (mg/dL)					
Liver	4.01 ± 0.32^a^	4.08 ± 0.27^a^	2.28 ± 0.21^b^	3.87 ± 0.24^c^	3.91 ± 0.26^c^
Kidney	3.26 ± 0.34^a^	3.36 ± 0.29^a^	1.89 ± 0.18^b^	2.93 ± 0.21^c^	2.99 ± 0.27^c^
Plasma	25.28 ± 3.25^a^	24.38 ± 3.76^a^	13.20 ± 2.17^b^	19.95 ± 2.81^c^	20.01 ± 2.98^c^

GLE: guava leaf extract. Values are given as means ± SD for six rats in each group. ^a^Group (group 2) with no significant difference compared to control group. ^b^Significantly different from control group at *p *< 0.05. ^c^Significantly different from diabetic group at *p *< 0.05. Duncan's Multiple Range Test (DMRT).

## Abbreviations

STZ: Streptozotocin
CAT: Catalase
CD: Conjugated dienes
DM: Diabetes mellitus
GB: Glibenclamide
SOD: Superoxide dismutase
GLE: Guava leaf extract
GSH: Reduced glutathione
IL-6: Interleukin-6
LOOH: Lipid hydroperoxides
NF-kB: Nuclear factor-kappa B
ROS: Reactive oxygen species
TBARS: Thiobarbituric acid reactive substances
TCA: Trichloroacetic acid
TBA: Thiobarbituric acid
TNF-*α*: Tumor necrosis factor-*α*.
